# *O*^6^-methylguanine-DNA-methyltransferase expression and gene polymorphisms in relation to chemotherapeutic response in metastatic melanoma

**DOI:** 10.1038/sj.bjc.6601270

**Published:** 2003-10-14

**Authors:** S Ma, S Egyházi, T Ueno, C Lindholm, E L Kreklau, U Stierner, U Ringborg, J Hansson

**Affiliations:** 1 Department of Oncology/Pathology, Cancer Centre Karolinska, Karolinska Hospital, S-171 76 Stockholm, Sweden; 2 Department of Oncology, Ryhov County Hospital, Jönköping, Sweden; 3 Indiana University Cancer Centre, Department of Pharmacology/Toxicology, Indiana University School of Medicine, Indianapolis, IN, USA; 4 Department of Oncology, Sahlgrenska University Hospital, Gothenburg, Sweden

**Keywords:** *O*^6^-methylguanine-DNA-methyltransferase, polymorphisms, melanoma, chemotherapy

## Abstract

In a retrospective study, *O*^6^-methylguanine-DNA-methyltransferase (MGMT) expression was analysed by immunohistochemistry using monoclonal human anti-MGMT antibody in melanoma metastases in patients receiving dacarbazine (DTIC) as single-drug therapy or as part of combination chemotherapy with DTIC–vindesine or DTIC–vindesine–cisplatin. The correlation of MGMT expression levels with clinical response to chemotherapy was investigated in 79 patients with metastatic melanoma. There was an inverse relationship between MGMT expression and clinical response to DTIC-based chemotherapy (*P*=0.05). Polymorphisms in the coding region of the *MGMT* gene were also investigated in tumours from 52 melanoma patients by PCR/SSCP and nucleotide sequence analyses. Single-nucleotide polymorphisms (SNPs) in exon 3 (L53L and L84F) and in exon 5 (I143V/K178R) were identified. There were no differences in the frequencies of these polymorphisms between these melanoma patients and patients with familial melanoma or healthy Swedish individuals. Functional analysis of variants *MGMT*-I143V and -I143V/K178R was performed by *in vitro* mutagenesis in *Escherichia coli*. There was no evidence that these variants decreased the MGMT DNA repair activity compared to the wild-type protein. All melanoma patients with the *MGMT* 53/84 polymorphism except one had tumours with high MGMT expression. There was no significant correlation between any of the *MGMT* polymorphisms and clinical response to chemotherapy, although an indication of a lower response rate in patients with SNPs in exon 5 was obtained. Thus, MGMT expression appears to be more related to response to chemotherapy than *MGMT* polymorphisms in patients with metastatic melanoma.

In recent decades, cutaneous malignant melanoma has shown a marked increase in incidence in developed countries with Caucasian populations. Disseminated melanoma is known to be an incurable disease that responds poorly to chemotherapy due to resistance to antitumour drugs. No regimen, so far, has demonstrated improved survival compared to single-agent therapy with dacarbazine (DTIC) in disseminated melanoma (Serrone *et al*, 2000). Thus, new insights into the mechanisms of resistance to chemotherapeutic drugs are essential, since they may lead to development of predictive tests that can identify individuals with tumours sensitive to a specific agent, as well as to the development of strategies to circumvent drug resistance, thereby improving the results of therapy.

Dacarbazine, which is commonly used in chemotherapy of metastatic melanoma, is a methylating cytostatic drug which produces *O*^6^-methylguanine (*O*^6^-mG). *O*^6^-methylguanine in DNA mis-pairs with thymine during DNA synthesis and initiates repeated ineffective cycles of DNA mismatch repair (MMR). This results in generation of DNA strand breaks, which may induce apoptotic signal transduction and thus kill the tumour cells (Pegg, 1990; Karran and Bignami, 1994).

*O*^6^-methylguanine-DNA-methyltransferase (MGMT) is a DNA repair protein which can transfer the methyl group from the *O*^6^-atom in the guanine base to an internal cysteine residue at codon 145 in the protein. This protein thus removes the primary cytotoxic lesion induced by *O*^6^-methylating agents, such as DTIC, thereby preventing cytotoxicity and causing resistance to the drug. *O*^6^-methylguanine-DNA-methyltransferase thus confers resistance to certain alkylating antitumour agents such as the methylating drugs DTIC and temozolomide (TMZ) in cultured tumour cells (Pegg, 1990, 2000), including melanoma cells (Lage *et al*, 1999). *O*^6^-methylguanine-DNA-methyltransferase knockout mice have been shown to be sensitive to the effects of chemotherapeutic alkylating agents (Glassner *et al*, 1999). Clinical studies of the relationship between MGMT levels and response to chemotherapy have given differing results in different tumour types (Friedman *et al*, 1998b; Middleton *et al*, 1998; Park *et al*, 2002). To some extent these differences may depend on the various techniques used to assay MGMT and tumour heterogeneity. We have previously shown that MGMT expression varies considerably in different melanoma tumours, both between patients and in the same patient (Egyházi *et al*, 1997; Ma *et al*, 2002).

Single-nucleotide polymorphisms (SNPs) represent an important class of genetic variants, and SNPs in genes encoding the enzymes responsible for drug metabolism are under intense investigation to define possible molecular differences of importance for drug metabolism and response to pharmacologic therapy. Single nucleotide polymorphisms in the *MGMT* gene can have effects on the MGMT activity (Hazra *et al*, 1997) or on sensitivity to the MGMT inhibitor *O*^6^-benzylguanine (*O*^6^-BG) (Edara *et al*, 1996; Pegg *et al*, 1998) and might therefore have an effect on clinical response to DTIC-based chemotherapy in melanoma.

In a previous study (Egyházi *et al*, 2002), we investigated SNPs in the promoter and coding regions of the *MGMT* gene in blood from patients with familial melanoma and healthy Swedish individuals. In total, 11 SNPs of the *MGMT* gene were identified in that study, including variants at codons 53 and 84 in exon 3, and at codons 143, 178 and 197 in exon 5. Codon 143 is very close to the MGMT active site cysteine-145, and the polymorphism at codon 143 appears to be linked to the codon 178 polymorphism. Owing to its location, it is possible that the 143 variant itself, or in combination with the 178 variant, might have an effect on MGMT activity.

In a previous study, we evaluated MGMT expression in human melanoma metastases, and saw a tendency of lower MGMT expression in responders to DTIC-based chemotherapy compared with nonresponders (Ma *et al*, 2002). In the present study, MGMT expression has been analysed in additional tumour samples to further investigate whether MGMT may be a drug resistance factor to DTIC-based chemotherapy in melanoma. Single nucleotide polymorphisms in the coding regions of the *MGMT* gene have also been analysed to investigate the possible relevance of *MGMT* SNPs for response to chemotherapy in melanoma patients. Functional analysis of the codon 143 variant and double variant 143/178 of *MGMT* was also performed by *in vitro* mutagenesis in *Escherichia coli*.

## MATERIAL AND METHODS

### Patients and tumour biopsies

Tumour biopsies from 79 patients with metastatic melanoma were included in this study, of whom 65 were included in our earlier study (Ma *et al*, 2002). Of these patients, 73 had been treated at Radiumhemmet, Karolinska Hospital with DTIC as single drug or had participated in a randomised phase III trial comparing DTIC in combination with vindesine to a three-drug regimen of DTIC, vindesine and cisplatin (Jungnelius *et al*, 1998). In addition, six patients had received chemotherapy with DTIC alone outside Karolinska Hospital (four patients from Ryhov Hospital in Jönköping, one from Sahlgrenska University Hospital in Gothenburg and one from Växjö Central Hospital). Clinical response data were obtained from patient records. WHO criteria for clinical response were used for classification in the categories complete response (CR), partial response (PR), stable disease (SD) and progressive disease (PD). Patients with CR and PR were grouped together as responders (R) and those with SD and PD as nonresponders (NR).

A total of 110 biopsies from melanoma metastases obtained before chemotherapy from 79 patients were investigated. In 20 of the patients, more than one metastasis was analysed. There were 47 male and 32 female patients with a median age of 58 years (range 22–83 years). In the whole group of 79 patients, 20 were classified as R and 59 as NR. Of the 53 patients who had received chemotherapy with DTIC alone, 12 patients were classified as R and 41 as NR.

### Immunohistochemistry

Immunohistochemical staining for MGMT was performed in the same manner as described previously (Ma *et al*, 2002). Briefly, monoclonal human anti-MGMT antibody (20 *μ*g ml^−1^, clone MT3.1, Chemicon International, Inc., Temecula, CA, USA) was used in the formalin-fixed, paraffin-embedded tissues, with mouse IgG1 as negative control. The tissue sections were incubated with a biotinylated universal second antibody and streptavidin/peroxidase complex (Vector Laboratories, Inc., Burlingame, CA, USA), exposed to 3,3-diaminobenzidine tetrahydrochloride (DAB) for chromogen development, and counterstained with haematoxylin.

For each biopsy, the whole slide was examined and the overall proportion of MGMT staining tumour cells was estimated regardless of intensity of staining. For comparison with clinical data on treatment outcome, the tumour biopsies were divided into two groups with <50% and ⩾50% tumour cells staining positively for MGMT. In cases where more than one tumour from the same individual had been analysed, with different outcome with respect to the proportion of MGMT-positive tumour cells, the tumour with the highest percentage of MGMT-positive cells was used for the comparison with outcome of clinical treatment. Statistical analyses were performed with the *χ*^2^ test or Fisher's exact two-tailed test.

### Genetic polymorphism analysis

DNA was extracted from the paraffin-embedded biopsies of melanoma metastases. The tumour cells were carefully microdissected from the selected tumour area with a scalpel from two or three 20 *μ*m sections guided by haematoxylin- and eosin-stained slides, since this material was also used to screen for somatic mutations in melanoma cells as part of another project. After deparaffinisation, the scraped tissue was suspended in digestion buffer containing 10 mM Tris-HCl, pH 8.3, 1 mM EDTA, 0.5% Tween-20 and 50 *μ*g ml^−1^ proteinase K and incubated overnight at 56°C in a shaker. DNA was purified by the Wizard DNA clean-up system (Promega Madison, WI, USA).

The extracted DNA was analysed by PCR/SSCP and nucleotide sequencing. The MGMT coding regions, exons 2–5, were amplified by PCR with exon-specific PCR primers (Otsuka *et al*, 1996; Wang *et al*, 1997). PCR reactions were performed on a Perkin-Elmer 9600 thermal cycler (Applied Biosystems, Foster City, CA, USA) under the following amplification conditions: denaturation at 94°C for 4 min, followed by 30 cycles at 94°C for 30 s (35 cycles 40 s for exon 4), annealing for exons 2, 3 and 5 at 66, 60, and 67 for 30 s, respectively (exon 4 at 55°C for 50 s), and 72°C for 30 s (exon 4 for 60 s), and then a 7-min extension. The PCR products were 210, 201, 184 and 295 bp, respectively. The PCR product of exon 5 was cleaved with *Sac*I before electrophoresis for optimal resolution on the SSCP gel. The PCR products, labelled by incorporation of [*α*-^32^P]dCTP, were denatured in denaturing buffer at 92°C for 10 min, followed by SSCP analysis carried out on 7.5% nondenaturing polyacrylamide gels in the presence of 10% glycerol at 14°C (exon 4), 18°C (exon 2) or 25°C (exon 3) and in the absence of glycerol at 18°C (exon 5). Finally, the dried gels were exposed to X-ray film. Samples that showed a motility shift pattern in the SSCP films were always subjected to a reanalysis starting from PCR amplification. Only those samples showing reproducible alterations were subjected to DNA sequencing.

Nucleotide sequencing was performed in both directions with the same primers as used in PCR using the Big Dye terminator method and an ABI 310 genetic analyzer (Applied Biosystems).

### *In vitro* mutagenesis in *E. coli* and analyses of protein expression and DNA repair activity

*MGMT* expressed in plasmid Bluescript II KS+ (pBS) was used for construction of *MGMT* variant isoleucine 143 to valine (I143V) and combined with variant lysine 178 to arginine (K178R), and the *MGMT* constructs expressed in plasmid pUC-18 was used in studies of MGMT expression and DNA repair activity in *E. coli*. *E. coli* mutant strain GWR111 (Δada-25::Cam^r^, Δogt-1:: Kan^r^) was used as methyltransferase-deficient host cells for expression of the pUC-*MGMT* cDNA constructs.

*MGMT* variants I143V and I143V/K178R were generated using the PCR-based overlap extension technique (Ho *et al*, 1989). To construct these variants, primers were designed as primer A (5′-AATCC*G*GT*A*CCCATCCTC GTCCCGT-3′) and primer B (5′-ATGGG*T*ACCGGATTGCCTCT-3′) for variant I143V, and primer C (5′-TTGGGGAGGCC*T*GGCTTGG-3′) and primer D (5′-TCCCTCCCAAGCC*A*GGCCT-3′) for variant K178R (mismatches underlined and *Asp*718 and *Stu*I sites shown in italics). To generate *MGMT* variant I143V, the PCR reaction was carried out using Pfu polymerase (Stratagene La Jolle, CA, USA) with pBS-*MGMT* plasmid as template and A and T7 or B and T3 as primers, respectively, under the following conditions: 30 cycles of denaturation for 45 s at 94°C, annealing for 45 s at 55°C, extension for 1 min at 72°C. The two halves of the cDNA were joined in a second PCR with T7 and T3 as primers. This second PCR was performed under the same conditions as described for the primary PCR. *MGMT* variant I143V/K178R was generated in the same manner except using pBS-*MGMT* I143V as template and primers C and D instead of primers A and B. The PCR product (∼1 kb) was gel-purified (Geneclean II: Bio101, La Jolla, CA, USA), digested by *EcoR*I (Roche Diagnostics Scand AB, Stockholm, Sweden), repurified and then ligated into the linearised and dephosphorylated vector pUC-18 to get the constructs pUC-*MGMT* variants I143V, I143V/K178R and *MGMT* wild-type, respectively. These constructs were introduced into *E. coli* strain GWR111 by chemical transformation. After selection of the white clones containing recombinant *MGMT* cDNA by a-complementation with X-gal and IPTG, plasmid DNA was isolated by JET quick plasmid miniprep kit (Genomed GmbH, Germany), and digested by either *EcoR*I and *Asp*718 or *EcoR*I and *Stu*I (Roche) to verify that the selected clones contained the constructs pUC-*MGMT* I143V and pUC-*MGMT* I143V/K178R, respectively. The selected plasmid DNA was digested by *Sac*I to select the subclone in the right insert direction, and was then sequenced to confirm the right insertions without any secondary variants.

Crude cell extracts for the MGMT expression and DNA repair activity analyses were prepared from different transformed *E. coli* grown to OD^600^ 0.6–0.8 in 2 ml LB medium with 50 *μ*g ml^−1^ ampicillin and 50 *μ*g ml^−1^ kanamycin by sonicating the bacterial pellet resuspended in TE buffer with 100 *μ*g ml^−1^ lysozyme and 1% Triton X-100 at 37°C for 10 min. Cell debris was pelleted and the supernatant was used to determine MGMT expression and DNA repair activity in *E. coli*. Protein amount in cell extracts was determined with Bio-Rad's protein assay using a BioSpec-1601E protein analyser (Shimadzu, Japan).

Studies of expression of I143V and I143V/K178R variant MGMT proteins was performed by Western blot analyses. Cell extract proteins (4 *μ*g) were resolved by 12% polyacrylamide–SDS gel electrophoresis along with a Rainbow™ coloured protein molecular weight marker (Amersham Pharmacia Biotech AB, Uppsala, Sweden) and transferred onto a polyvinylidene difluoride membrane for Western blotting. The clone MT3.1 monoclonal MGMT antibody was used at the concentration of 1 *μ*g ml^−1^ (1 : 1000). HRP-conjugated anti-mouse antibody (Rockland, PA, USA) was incubated at a concentration of 1 : 10 000. The membrane was developed with SuperSignal West Pico chemiluminescence substrate (Pierce, Rockford, IL, USA) and exposed to X-ray film. *O*^6^-methylguanine-DNA-methyltransferase expression was quantified by a MultiImager (BioRad Hercules, CA, USA).

The MGMT activity analyses were performed as described previously (Wu *et al*, 1987; Kreklau *et al*, 1999) with some modification. Briefly, an 18-bp oligomer was synthesised to contain the *O*^6^-mG lesion within a *Pvu*II restriction site. This oligo was radiolabelled by filling in the 3′ recessed end of the complementary 16-bp strand with [*α*-^32^P] dTTP, followed by purification with QIAquick nucleotide removal kit (Qiagen GmbH, Hilden, Germany) to remove the incorporated radioactive probe. *O*^6^-methylguanine-DNA-methyltransferase activity was measured by incubating 0.2 pmol of the radiolabelled probe with 1 and 5 *μ*g of cell extract protein at 37°C for 2 h. The probe was then digested with *Pvu*II (Roche) and electrophoresed on a 20% denaturing polyacrylamide gel. Results were quantified on a MultiImager. MGMT activity is proportional to the amount of radiolabelled 8-bp fragment produced.

For the *N*-methyl-*N*′-nitro-*N*-nitrosoguanidine (MNNG) survival assay, GWR111-containing plasmids expressing *MGMT* wild-type, variants I143V, I143V/K178R or empty vector were grown in LB medium containing 50 *μ*g ml^−1^ ampicillin and 50 *μ*g ml^−1^ kanamycin with agitation at 37°C until the OD^600^ was 0.7. The cultures were then pelleted, washed, resuspended in LB medium and exposed to MNNG (0–40 *μ*g ml^−1^) for 20 min. The reactions were stopped by diluting small aliquots of bacterial cultures in LB medium on ice. The bacteria were further diluted and spread on LB plates containing the same antibiotics as in culture. The plates were incubated at 37°C for 16 h, and the numbers of colonies were counted. The percentage of survival was determined by calculating the ratios of colony numbers in cultures exposed to MNNG and those in cultures without treatment.

## RESULTS

Analysis of MGMT expression in biopsies of melanoma metastases was performed by immunohistochemistry using the MT 3.1 monoclonal human anti-MGMT antibody. *O*^6^-methylguanine-DNA-methyltransferase expression varied considerably between tumours from different melanoma patients and also among different metastases from the same patient, ranging from completely negative to 100% positive staining. Among all 79 patients, 65 of whom were included in our earlier study (Ma *et al*, 2002), there were nine responders (41%) in 22 patients with tumours with low MGMT expression, while there were 11 responders (19%) in 57 patients carrying tumours with high expression of MGMT. This difference in response rate is statistically significant (*P*=0.05, *χ*^2^ test, Table 1a

**Table 1:** MGMT expression and clinical response to DTIC-based chemotherapy in 79 patients with metastatic melanoma

	**MGMT-positive cells**	
**Response to chemotherapy**	**<50%**	**⩾50%**
*All patients* (*n*=*79*) (*P*=*0.05* (*χ*^*2*^ *test))*		
Responders (*n*=20)	9 (41%)	11 (19%)
Nonresponders (*n*=59)	13 (59%)	46 (81%)
Response to DTIC	MGMT-positive cells	
	<50%	⩾50%
*Patients treated with DTIC only* (*n*=*53*) (*P*=*0.09* (*χ*^*2*^ *test))*		
Responders (*n*=12)	5 (42%)	7 (17%)
Nonresponders (*n*=41)	7 (58%)	34 (83%)

). Among 53 patients treated with DTIC alone, there was a similar tendency of improved clinical response in patients whose tumours had low MGMT expression. However, this difference was not significant (*P*=0.09, *χ*^2^ test, Table 1b), possibly due to the low number of patients treated with DTIC alone. Of the 79 patients analysed, nine obtained CR following chemotherapy. Of these, six had low MGMT expression (<50%). However, among the six CRs to single-agent DTIC therapy three patients had tumours with high MGMT protein levels (⩾50%), indicating that other factors than MGMT may influence clinical response. In 20 of the patients more than one metastasis was analysed, and in nine of these cases the MGMT expression differed between tumours in the same individual according to the 50% cutoff level.

Single nucleotide polymorphisms in the coding regions of the *MGMT* gene, exons 2–5, were investigated in DNA extracts from melanoma metastases by PCR/SSCP and nucleotide sequencing. The variants were found in exons 3 and 5 of the *MGMT* gene (Table 2

**Table 2:** Frequency of MGMT SNPs in patients with metastatic melanoma compared with the results of our earlier study of familial melanoma and Swedish healthy subjects

**Gene region**	**Nucleotide alteration**	**Coding effect**	**Functional effect *in vitro***	**Metastatic melanoma (*n*=52)**	**Familial melanoma (*n*=89)**	**Healthy subjects (*n*=76)**
Exon 3	Codon 53 CTC>CTT	Leu >Leu silent	None (?)	8 (15%)	17 (19%)	16 (21%)
	Codon 84 CTT>TTT	Leu >Phe	None (Inoue, 2000)	9 (17%)	20 (23%)	19 (25%)
						
Exon 5	Codon 143 ATC>GTC	Ile >Val	None (this study)	11 (21%)	21 (24%)	17 (22%)
	Codon 178 AAG>AGG	Lys >Arg	None (this study)	11 (21%)	21 (24%)	17 (22%)

). SSCP analysis of exon 3 of the *MGMT* gene revealed two band shift patterns: wt/53/84 and wt/84. The codon 53 variant was a silent alteration of L (CTC) to L (CTT). The variant of codon 84 was a missense alteration that converts L (CTT) to F (TTT). These codon 53 and 84 polymorphisms were first described in Japanese population by Otsuka *et al* (1996). In most cases (eight out of nine), the L84F and L53L were linked, although in one case only the L84F appeared. Our SSCP analysis of exon 5 in the *MGMT* gene showed a single band shift pattern: wt/143/178. The codon 143 variant was a change from I (ATC) to V (GTC), and the variant at codon 178 was from K (AAG) to R (AGG). The polymorphisms of codons 143 and 178 were always linked together. This finding has also been reported by Deng *et al* (1999) and by us in a previous study (Egyházi *et al*, 2002). No polymorphisms were found in exons 2 and 4 of the *MGMT* gene in our melanoma patients.

When the frequencies of *MGMT* SNPs in exons 3 and 5 in 52 melanoma metastases were compared to *MGMT* SNPs in members of melanoma families and Swedish healthy subjects, there were no significant differences (Table 2).

We studied the relation between *MGMT* SNPs and clinical response to DTIC-based chemotherapy in 52 melanoma patients (Table 3

**Table 3:** Frequency of MGMT SNPs and clinical response to DTIC-based chemotherapy in 52 patients with metastatic melanoma

	**MGMT SNPs**		
**Response to chemotherapy**	**Exon 3**	**Exon 5**	**Exon 3 and/or 5**
Responders (*n*=20)	4 (44%)	2 (18%)	5 (29%)
Nonresponders (*n*=32)	5 (56%)	9 (82%)	12 (71%)

). In patients with the exon 3 SNPs, the response rate was 44% (four out of nine) compared to 37% (16 out of 43) in those without these SNPs (*P*=0.69). In patients with the exon 5 SNPs, the response rate was 18% (two out of 11) compared to 44% (18 out of 41) in those without the SNPs (*P*=0.10). In patients with both exons 3 and 5 SNPs, the response rate was 29% (five out of 17) compared to 43% (15 out of 35) in those lacking either of the SNPs (*P*=0.34). There were thus no significant differences between patients with MGMT SNPs regard to clinical response to chemotherapy, although there was a tendency towards a lower response rate in patients with exon 5 SNPs. We also compared *MGMT* SNPs to MGMT protein expression, analysed by immunohistochemistry, in these 52 patients (Table 4

**Table 4:** MGMT SNPs and its expression by immunohistochemistry in 52 patients with metastatic melanoma

	**MGMT SNPs**					
**MGMT**	**Exon 3**		**Exon 5**		**Exon 3 and/or 5**	
**expression**	**SNPs+**	**SNPs−**	**SNPs+**	**SNPs−**	**SNPs+**	**SNPs−**
<50%	1 (11%)	14 (33%)	4 (36%)	12 (29%)	4 (24%)	11 (31%)
⩾50%	8 (89%)	29 (67%)	7 (64%)	29 (71%)	13 (76%)	24 (69%)

). In patients with exon 3 SNPs, a larger proportion of tumours with high MGMT expression was 89% (eight out of nine) compared to 67% (29 out of 43) without these SNPs (*P*=0.19, Fisher's test). A similar proportion was shown in either exon 5 or both these exons with or without the SNPs.

**Figure 1:**
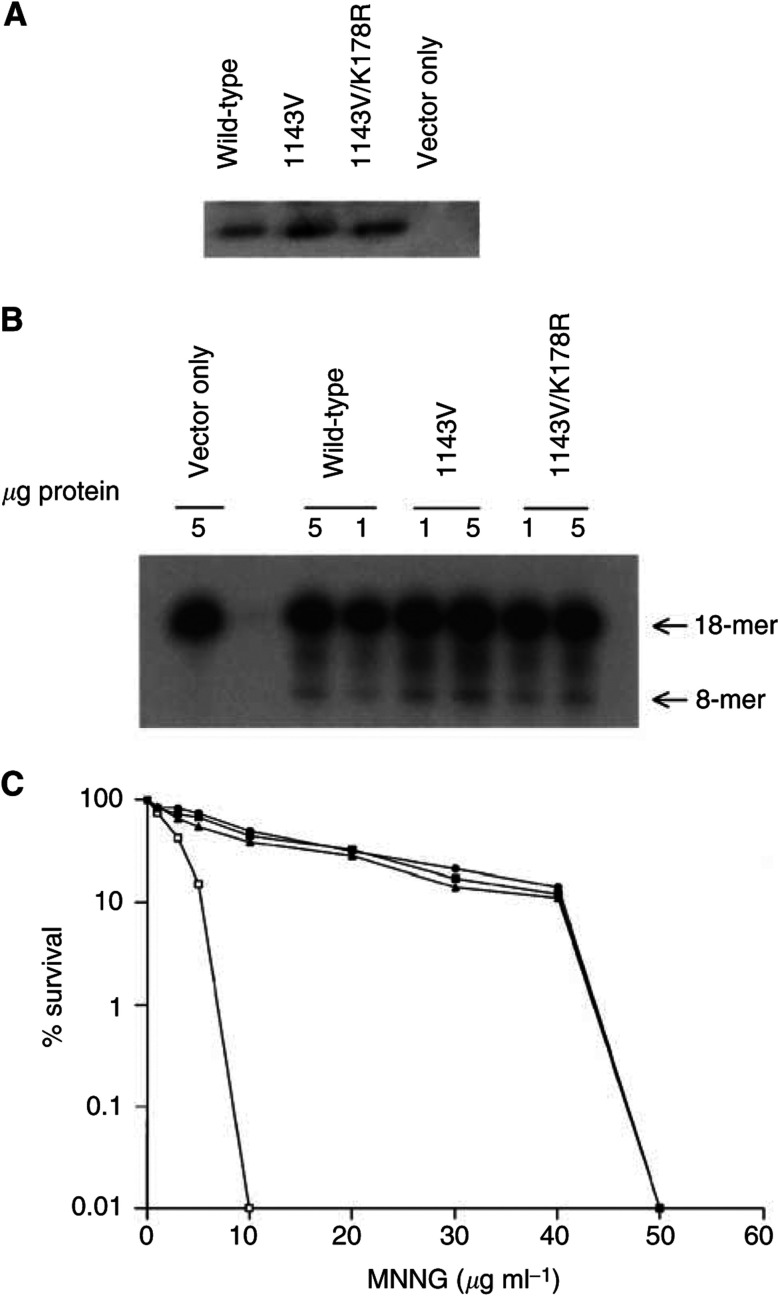
(**A**) Expression of variant MGMT protein in GWR111 cells. Extracts from GWR111 cells expressing wild type, its variants I143V, I143V/K178R or vector control were resolved by SDS–PAGE, transferred to membrane and developed using human anti-MGMT monoclonal antibody. (**B**) MGMT activity in GWR111 cells using ^32^P-labelled oligonucleotide. Extracts from GWR111 cells expressing vector control, wild type, its variants I143V or I143V/K178R were incubated with ^32^P-labelled oligonucleotide and cleaved with *Pvu*II. A measure of 1 or 5 *μ*g of protein were incubated. (**C**) The effects of variants I143V and I143V7K178R on the survival of MNNG-treated GWR111 cells. The survival of GWR111 cells expressing MGMT is shown after treatment with MNNG concentration. Results are shown for cells expressing wild-type MGMT (▪), variant I143V (▴), I143V/K178R (•), and vector control (□).

Functional analysis of variants I143V and I143V/K178R of *MGMT* was performed by *in vitro* mutagenesis in *E. coli*. Construction of *MGMT* variant pUC-I143V and double variant pUC-I143V/K178R was made by the PCR-based overlap extension technique, and these constructs were used to transform the MGMT-deficient *E. coli* strain GWR 111. Analysis of MGMT expression in *E. coli* was performed by Western blot with monoclonal anti-MGMT antibody. As shown in Figure 1A

, MGMT expression was not decreased in *E. coli* transformed with either variant I143V or I143V/K178R compared to wild-type *MGMT*. *O*^6^-methylguanine-DNA-methyltransferase DNA repair activity was tested with an oligomer containing *O*^6^-mG, which was incubated with 1 and 5 *μ*g of cell extract protein (Figure 1B). No reduced MGMT activity could be seen for either variant compared with wild-type protein. In fact, a marginally increased MGMT activity was shown for variant I143V. Analysis of MGMT function was also performed with an MNNG survival assay (Figure 1C). The sensitivity to MNNG in *E. coli* with *MGMT*-I143V or -I143V/K178R was at the same level as in *E. coli* with *MGMT* wild-type. As expected, *E. coli* lacking MGMT expression is considerably more sensitive to MNNG compared to bacteria expressing wild-type or variant *MGMT*.

## DISCUSSION

We observed an inverse relationship between MGMT expression and clinical response to DTIC-based chemotherapy in patients with metastatic melanoma (*P*=0.05), indicating that MGMT is a factor that contributes to drug resistance against DTIC-based chemotherapy in melanoma. It has been demonstrated previously in an extensive series of preclinical and clinical studies that MGMT is responsible for resistance to methylating agents (Pegg, 1990, 2000). A recent clinical trial showed that analysis of MGMT expression could predict resistance to TMZ in malignant glioma (Friedman *et al*, 1998b). The resistance to TMZ in renal cell cancer may also be due to high MGMT activity since high MGMT activity was observed in pretreatment biopsies from renal cell cancer from four patients who showed no response to TMZ (Park *et al*, 2002). In contrast, Middleton *et al* (1998) found no correlation between MGMT activity in tumour extracts and the clinical response to TMZ in metastatic melanoma. In that investigation, the MGMT activity measurements were performed on extracts from a single metastasis in each patient without regard to possible tumour heterogeneity. In fact, a marked heterogeneity of MGMT expression in melanoma tumour cells has been shown in our previous (Egyházi *et al*, 1997; Ma *et al*, 2002) and present studies. Different metastases in the same patient also frequently expressed different levels of MGMT, which may explain why some melanoma patients obtain only a partial response to chemotherapy even though they have a low MGMT expression in the excised tumours. Inter- and intratumour heterogeneity in expression of MGMT in different kinds of tumours seems to be common (Egyházi *et al*, 1997; Clemons *et al*, 2002; Lees *et al*, 2002; Ma *et al*, 2002). The heterogeneity between and within tumours from the same patient may be due to tumour cell subpopulations with differences in MGMT expression, possibly related to heterogeneity in MGMT promoter methylation. The variability in MGMT expression showed no correlation to proliferation when expression of Ki67 was analysed (Egyházi *et al*, 1997). Further investigations are required to clarify the causes of MGMT heterogeneity.

Some of our melanoma patients had been treated with combination chemotherapy of DTIC–vindesine–cisplatin, which complicates the evaluation. Cisplatin has been shown to decrease MGMT activity in a time- and dose-dependent manner in a human leukaemic cell line, and combination of TMZ and cisplatin caused substantial and prolonged MGMT depletion, suggesting that TMZ and cisplatin in combination may improve the clinical efficacy (D'Atri *et al*, 2000).

Recent reports have given insights into the complex mechanisms of regulation of MGMT expression. There are several different factors of importance for regulation of MGMT expression. p53 accumulation results in loss of MGMT mRNA and protein, due to a reduction in the rate of *MGMT* gene transcription. Thus, p53 is a negative regulator of *MGMT* gene expression which can create an MGMT-depleted state in human tumours (Srivenugopal *et al*, 2001). Expression of wild-type p53 was associated with low MGMT level in primary ovarian cancer, supporting the view that downregulation of basal *MGMT* promoter activity by p53 wild type is also relevant in tumour cells *in vivo* (Hengstler *et al*, 1999). However, in another study p53 has been shown to upregulate the MGMT expression (Rafferty *et al*, 1996). Other factors, such as a tyrosine kinase and a serine/threonine kinase phosphorylate the MGMT protein and thereby affect its function (Mullapudi *et al*, 2000). Histone acetylation also regulates MGMT expression (Bhakat and Mitra, 2000). Besides that, the methylation status of the *MGMT* gene has been demonstrated to have an impact on drug response. Acquired resistance to the chloroethylating antineoplastic agent fotemustine in melanoma cells is caused by reactivation of the DNA repair gene *MGMT*, which is associated with hypermethylation of the body of the gene (Christmann *et al*, 2001). In our study, all except one of the melanoma patients with the 53/84 polymorphism in exon 3 had tumours with high MGMT expression. One possible hypothesis explaining this phenomenon is that the methylation pattern in the body of the *MGMT* gene may be increased by these SNPs. Conversely, inactivation of MGMT by methylation of the promoter has been associated with clinical response of gliomas to alkylating agents (Esteller *et al*, 2000).

We also investigated polymorphisms of the *MGMT* gene in patients with metastatic melanoma in order to explore the possible role of these polymorphisms in DTIC-based chemotherapy. From our clinical data, there was no significant correlation between polymorphisms in exons 3, 5 or both and clinical response to DTIC-based chemotherapy. However, an indication was obtained of poorer response in patients with SNPs in exon 5, which fits well with the tendency to an increased MGMT activity in *E. coli* transformed with the exon 5 I143V variant. Thus, MGMT expression seems to be more relevant for response to chemotherapy than these *MGMT* SNPs. The SNPs in the *MGMT* gene identified in these melanoma patients are heterozygous; thus the wild-type allele is always present. It is therefore of interest to study whether the gene variant is expressed in those tumours, to be able to draw definite conclusions regarding its effect on protein activity.

These polymorphisms identified in our melanoma patients may affect MGMT function differently. We have assumed that the codon 53 silent polymorphism has no effect on MGMT activity, but this may be wrong. As mentioned before, this SNP might possibly have an effect on the methylation status of the *MGMT* gene and thereby have an impact on MGMT expression and activity. The codon 84 polymorphism of the *MGMT* gene present in Swedish individuals (Egyházi *et al*, 2002), and also seen in Japanese (Otsuka *et al*, 1996), Chinese and other Caucasian populations (Deng *et al*, 1999), has no defect MGMT function (Inoue *et al*, 2000). The codon 178 polymorphism is unlikely to affect MGMT activity since MGMT can be truncated at position 176 with no loss of activity, but this SNP may still affect the protein's stability (Hazra *et al*, 1997). Codon 143 is very close to the active site cysteine-145 of MGMT, and thus the codon 143 SNP could have an effect on MGMT activity. We therefore performed a functional analysis by *in vitro* mutagenesis in *E. coli* to investigate whether the *MGMT* I143V or I143V/K178R variants could have an effect on MGMT activity. *E. coli* strain GWR111 carrying variant *MGMT* I143V or double variant I143V/K178R exhibited almost the same identical sensitivity against MNNG as did GWR111 with wild-type *MGMT*, and assays of MGMT expression and MGMT activity showed no decrease compared to the wild-type protein. No evidence was shown that variants *MGMT* I143V and I143V/K178R have a negative effect on the MGMT activity in *E. coli*.

Our data indicate that MGMT expression had a significant correlation to clinical response, suggesting that MGMT may be an important drug resistance factor against DTIC-based chemotherapy in patients with metastatic melanoma. *O*^6^-methylguanine-DNA-methyltransferase may thus be used as a target to sensitise tumour cells to improve the clinical efficacy in patients with chemotherapy-resistant tumours. Inhibitors of MGMT activity, such as *O*^6^-BG are now investigated in clinical trials. Phase I studies of *O*^6^-BG have already been carried out with the aim of modulating MGMT activity and thereby circumventing drug resistance in the clinic (Friedman *et al*, 1998a; Friedman *et al*, 2000; Schilsky *et al*, 2000). A phase II trial of *O*^6^-BG plus BCNU for chemotherapy-resistant tumours has been conducted and none of the 18 participating patients showed any response although stable disease was seen in five patients (Quinn *et al*, 2002). Another MGMT inhibitor, *O*^6^-4-bromothenyl-guanine (4BTG) enhanced the antitumour effect of TMZ in human melanoma xenografts (Middleton *et al*, 2000). The use of such inhibitors may help to determine the role of MGMT in resistance to DTIC-based chemotherapy and may be used to improve the clinical results in treatment of malignant melanoma. A future approach to treatment of tumours with high expression of MGMT may consist of depletion of tumour MGMT with *O*^6^-BG and protection of sensitive bone marrow cells, using genetic modification with *O*^6^-BG-resistant *MGMT* mutants, such as P140 K and G156A. P140 K *MGMT* gene transfer in a murine model indicates significant resistance to the myelosuppressive effects of TMZ and *O*^6^-BG (Sawai *et al*, 2001), and P140A MGMT protects haematopoietic cells against *O*^6^-BG sensitisation to chloroethylnitrosourea treatment (Maze *et al*, 1999). G156A *MGMT* mutant cDNA has been transducted into haematopoietic progenitors, resulting in remarkable resistance to *O*^6^-BG and BCNU (Davis *et al*, 1997). A clinical trial involving transduction of G156A *MGMT* into CD34+ cells of patients with cancer has been approved (Koc *et al*, 1999).

In conclusion, we found a significant relationship between MGMT expression and clinical response to DTIC-based chemotherapy. Owing to tumour heterogeneity, a possible future strategy to obtain a more correct evaluation of MGMT levels in relation to clinical response to DTIC-based treatment is to analyse multiple tumours in patients using fine-needle biopsies. This could help to determine to what extent MGMT has a clinical impact as a drug resistance factor in malignant melanoma. The fact that only 50% of the responders with single-agent DTIC therapy had tumours with <50% MGMT-positive tumour cells, indicates that other additional factors are involved in response to chemotherapy in these melanoma patients. Analysis of a panel of multiple potential factors should be performed by gene expression profiling in melanoma patients to find new potential factors related to resistance or sensitivity to chemotherapy.
